# Exhausted and Senescent CD4^+^ T Cells in Peripheral Blood and Their Impact as a Biological Marker for the Diagnosis of Benign and Malignant Ovarian Tumors

**DOI:** 10.3390/diagnostics15162019

**Published:** 2025-08-12

**Authors:** Gabriela Nohemi Espinoza-de-León, Adriana Aguilar-Lemarroy, Alejandra Natali Vega-Magaña, Ana Laura Pereira-Suarez, José Francisco Muñoz-Valle, Raquel Villegas-Pacheco, Luis Felipe Jave-Suárez, Mariel García-Chagollán

**Affiliations:** 1 Instituto de Investigación en Ciencias Biomédicas, Centro Universitario de Ciencias de la Salud, Universidad de Guadalajara, Guadalajara 44340, Mexico; nohemi_90@hotmail.com (G.N.E.-d.-L.); biologiamolecular@hotmail.com (J.F.M.-V.); 2 División de Inmunología, Centro de Investigación Biomédica de Occidente, Instituto Mexicano del Seguro Social, Guadalajara 44340, Mexico; adry.aguilar.lemarroy@gmail.com; 3 Laboratorio de Investigación en Cáncer e Infecciones, Centro Universitario de Ciencias de la Salud, Universidad de Guadalajara, Guadalajara 44340, Mexico; alejandra.vega@academicos.udg.mx (A.N.V.-M.); ana.pereira@academicos.udg.mx (A.L.P.-S.); 4 Unidad Médica de Alta Especialidad, Hospital de Ginecología y Obstetricia, Centro Médico Nacional de Occidente, Instituto Mexicano del Seguro Social, Guadalajara 44340, Mexico; villegas.raquel@icloud.com

**Keywords:** ovarian tumors, exhausted T cells, senescent T cells, ROMA

## Abstract

**Introduction:** Serum biomarkers such as CA-125 and HE4, along with the ROMA score, (which integrates both markers) are widely used to distinguish between benign and malignant ovarian tumors. In ovarian cancer, chronic exposure to tumor-associated antigens (TAAs), such as CA-125 and HE4, can lead to T cell exhaustion and senescence, thereby facilitating immune evasion. This study aimed to evaluate exhausted and senescent T cells in the peripheral blood of patients with benign or malignant ovarian tumors, and compare these findings to those of healthy donors, and assess their correlation with the ROMA score. **Methods:** The expression of senescent and exhaustion markers was evaluated on peripheral CD4^+^ T cells from patients with benign and malignant ovarian tumors, as well as healthy donors. Multicolor flow cytometry was performed to evaluate the expression of CTLA-4, PD-1, Tim3, CD28, CD57, and CD27. **Results:** PD1^+^Tim3^+^CD4^+^ expression was significantly higher in the malignant group compared to both the benign group (*p* = 0.05) and healthy donors (*p* = 0.015). A positive and significant correlation was observed between ROMA and PD-1^+^Tim3^+^ T cells (r = 0.44, *p =* 0.0006). The confusion matrix demonstrated good classification accuracy, and in the ROC analysis, the combination of ROMA and PD-1^+^Tim3^+^ yielded the highest Youden Index (0.75) and superior specificity (88.8%) compared to ROMA alone, albeit with a slight reduction in sensitivity (86.9% vs. 91.3%). A nomogram integrating ROMA and PD-1^+^Tim3^+^ exhibited strong predictive performance, with a concordance index (C-index) of 0.91. **Conclusion:** The combination of the ROMA score with the expression of PD-1^+^ and Tim-3^+^ in CD4^+^ T cells creates a simple yet highly effective model to differentiate between benign and malignant ovarian tumors.

## 1. Introduction

Ovarian cancer (OC) is one of the most lethal malignancies affecting women worldwide. This malignancy represents a significant global health concern, with notable variations in incidence and mortality across different regions. Because its early symptoms are nonspecific and easily mistaken for other conditions, its diagnosis is frequently made at advanced stages. Therefore, it is often referred to as the “silent killer” [1]. OC is classified based on the type of cells from which it originates. The most common type, epithelial ovarian cancer (EOC), accounts for approximately 90% of all cases of OC and includes high-grade and low-grade serous carcinoma, mucinous carcinoma, endometrioid carcinoma, and clear cell carcinoma. Other types include Germ Cell Tumors and Sex Cord-Stromal Tumors [1].

In 2020, a total of 313,959 women were diagnosed with OC worldwide, and approximately 207,252 deaths were reported [2,3]. By 2040, new cases are expected to increase by 42% to 445,721 annually, highlighting a growing global concern. In Mexico, OC is the sixth leading cause of cancer-related deaths, with 5193 new cases and 3360 deaths reported in 2020, reflecting a mortality rate of 4.0 per 100,000 women [2,3].

Currently, there is no screening test for the early-stage detection of OC. In women with a pelvic mass, diagnosing OC involves distinguishing between malignant and benign tumors. The diagnostic approach involves a three-stage evaluation: ultrasound imaging, the assessment of the patient’s menopausal status, and the evaluation of tumor markers [4,5]. The tumor markers that show particular relevance to improving the clinical management of OC include the cancer antigen 125 (CA125) and the human epididymis secretory protein 4 (HE4) [6]. Although CA-125 and HE4 are upregulated in most EOC, their expression can also be influenced by different factors such as age, menstrual cycle, smoking, renal failure, inflammatory diseases of the peritoneum, and other malignancies [7,8,9]. The combination of CA-125, HE4, and menopausal status has been used to calculate a risk score called ROMA (Risk of Ovarian Malignancy Algorithm). The ROMA score helps determine if a woman with a pelvic mass is at a high or low risk of having a malignant tumor [9]. Although ROMA has high sensitivity, it lacks high specificity [10,11]. Therefore, additional markers are needed, with alterations in the immune system being an interesting area to explore.

The immune system plays an important role in cancer prevention by identifying and eliminating abnormal cells before they develop into tumors. T cell-mediated antitumor immunity serves as a vital defense mechanism against the development of cancer. T cells recognize tumor-associated antigens presented on major histocompatibility complex (MHC) molecules, undergo activation and clonal expansion, and subsequently kill transformed cells employing different effector molecules [12]. However, cancer cells can evade immunosurveillance by creating an immunosuppressive microenvironment, leading to dysfunctional T cell states. Exhaustion and senescence are two major dysfunctional states of T cells, particularly evident in cancer [13]. Both phenotypes share certain functional features, such as reduced proliferative capacity, cell cycle arrest, and decreased cytokine secretion. Although dysfunctional T cells retain the ability to recognize antigens, they fail to mount an effective response against tumor cells.

Exhausted T cells arise due to chronic antigen exposure, such as tumor-associated antigens in cancer, resulting in diminished functionality and impaired tumor targeting [14]. These cells express inhibitory receptors like PD-1 and CTLA-4, which suppress their activity and enable tumor immune evasion [15,16]. In contrast, senescent T cells are aged, dysfunctional immune cells that accumulate over time, weakening immune function and promoting cancer progression [13,17]. They exhibit distinct phenotypic changes, including the absence of costimulatory molecules (CD27 and CD28), reduced chemokine receptor expression (CCR7 and CD45RO), and increased expression of senescence-associated markers such as TIM-3, CD57, CD45RA, and KLRG-1 [18].

Despite being antigen-specific, both exhausted and senescent T cells are unable to proliferate upon T cell receptor (TCR) stimulation due to downregulated TCR signaling components [16,19]. The frequency, mechanisms, and targeting of these dysfunctional T cell populations are crucial for improving cancer diagnosis and for developing therapies to enhance the body’s natural ability to fight cancer [20].

TAAs in OC may also contribute to T cell dysfunction, thereby facilitating immune evasion. Chronic exposure to TAAs can drive T cell exhaustion and the accumulation of senescent T cells, further impairing antitumor immunity [21,22]. While bolstering Treg regulatory activity, the chronic antigen stimulation in tumors or persistent viral infections drives elevated inhibitory receptor expression by inducing transcription factors [23]. In regulatory T cells, FOXP3 orchestrates transcriptional programs upregulating co-inhibitory receptors including CTLA-4, TIGIT, and PD-1 [24]. Cell–cell interactions amplify these effects; CTLA-4 on Tregs binds with CD80/CD86 on antigen-presenting cells with higher affinity than CD28, resulting in trogocytosis and costimulatory ligand removal, while increasing local inhibitory receptor concentrations [25].

The presence of these dysfunctional T cell populations may mark the transition from benign to malignant ovarian tumors, suggesting their potential role in disease progression. The aim of the present study was to evaluate the frequency of exhausted and senescent T cells in peripheral blood from patients with benign or malignant ovarian tumors, compare these frequencies with those from healthy donors, and ultimately correlate our findings with the ROMA score to assess their clinical relevance.

## 2. Materials and Methods

### 2.1. Human Samples and Peripheral Blood Collection

A total of 74 women were recruited for this study, among them 25 patients were diagnosed with malignant ovarian tumors and 31 patients with benign ovarian tumors, and 18 women were non-cancer healthy donors. The eligibility criteria for inclusion were age between 18 and 75 years, no ongoing infections, no autoimmune diseases, and no other types of cancer. This study was approved (register number: R-2023-1305-008) by the scientific and ethics committee (CLIS-1305) from Centro de Investigación Biomédica de Occidente, and all the study subjects provided written informed consent. The malignant ovarian tumors were confirmed to be of epithelial origin. Tumor classification into benign or malignant categories was based on a combined assessment including macroscopic examination, hematoxylin and eosin (H&E) staining, microscopic analysis, and, in the case of malignant tumors, immunohistochemical evaluation when necessary. All pathological assessments were performed by a board-certified pathologist following standard diagnostic criteria. All diagnosis, subject recruitment, and sample collection of the patients with ovarian tumors were carried out at the Unidad Médica de Alta Especialidad: Hospital de Ginecología y Obstetricia (HGO) from Centro Médico Nacional de Occidente (CMNO) of Instituto Mexicano del Seguro Social (IMSS). Peripheral blood samples were collected before surgery (from patients with benign and malignant ovarian tumors) and from healthy donors after an interview and pelvic ultrasound. Peripheral blood mononuclear cells (PBMCs) were isolated by centrifugation using LymphoPrep ™ (Stemcell Technologies, Seattle, WA, USA), washed twice with phosphate-buffered saline (PBS, 0.15 M, pH 7.2), counted, and then washed again with RPMI-1640 medium (Gibco, Thermo Scientific, Waltham, MA, USA). Afterward, the samples were resuspended in RPMI-1640 medium supplemented with 10% dimethyl sulfoxide (DMSO, Sigma-Aldrich, Burlington, MA, USA) and 20% fetal bovine serum. The cells were gradually frozen at −80 °C and cryopreserved in the gas phase of liquid nitrogen until use.

### 2.2. Staining and Flow Cytometric Analysis

The cells were thawed at 37 °C and washed twice with PBS. Afterward, 5 × 10^5^ cells were stained with fluorochrome-labeled monoclonal primary antibodies: CD3-FITC (clone: UCHT1), CD4-PerCP/Cy5.5 (clone: RPA-T4), CD8-BV510 (clone: SK1), CD27-PE/Cy7 (clone: O323), PD-1-Alexa Fluor 700 (clone: EH12,2H7), CD57-BV421 (clone: QA17A04), CTLA-4-PE (clone: BN13), CD28-APC (clone: CD28.2), CD45RO-BV605 (clone: UCHL1), NKG2D-APC/Cy7 (clone: 1D11), and Tim-3-BV711 (clone: F38-2E2) (all from BioLegend, San Diego, CA, USA). Samples were incubated for 30 min in the dark at 4 °C and then washed before acquisition on an Attune NxT flow cytometer. Data were analyzed using the FlowJo v10 software.

### 2.3. Serum CA125 and HE4 Measurement Using Commercial ELISA Kits

The serum levels of CA125 and HE4 were quantified using ELISA kits from R & D Systems (Cat. Nos. DCA125 and DE400, respectively), following the manufacturer’s instructions. All samples, standards, and controls were assayed in duplicate. Briefly, 50–100 µL of assay diluent and an equal volume of sample, standard, or control were added to each well. Plates were incubated at room temperature for 2 h, either with or without orbital shaking, depending on the assay. Wells were washed four times, and 200 µL of conjugate was added, followed by another 2 h incubation under the same conditions. After a second washing step, 200 µL of substrate solution was added and incubated for 30 min at room temperature, protected from light. The reaction was stopped with 50 µL of stop solution, and absorbance was measured at 450 nm with wavelength correction at 540 nm within 30 min.

### 2.4. Statistical Analysis

The normality of the data was assessed using the Shapiro–Wilk test. For variables that followed a normal distribution, ANOVA was used for the overall comparison among the groups. Tukey’s post hoc test was applied to pairwise comparisons between groups. Non-parametric tests were used for variables that did not follow a normal distribution. Kruskal–Wallis test was used to compare differences among the three groups, and Mann–Whitney U test was applied as a post hoc analysis to identify pairwise differences between groups when the overall Kruskal–Wallis test was significant. Statistical analyses were performed in R (version 4.4.2) within the RStudio environment (version 2024.12.0.467), and graphical visualizations were generated using the ggplot2 package (version 3.5.1).

## 3. Results

### 3.1. Characteristics of the Study Population

This study included 56 patients (31 diagnosed with benign tumors and 25 diagnosed with malignant tumors) and 18 healthy donors. The clinical characteristics and biomarkers are shown in Table 1. Age was significantly different among the groups (*p* = 0.012), specifically higher in the malignant group compared to the healthy donors (*p* = 0.03). BMI was significantly different between the groups (*p* = 0.004), specifically between benign tumors and healthy donors and malignant tumors and healthy donors (*p* = 0.004 and *p* = 0.02, respectively). The serum levels of CA-125 and HE4 were significantly higher in the malignant tumors compared to the benign and healthy donors (*p* < 0.0001).

### 3.2. Comparison of the Proportion of CD4^+^ T Cells Among Study Groups

**Figure 1:**
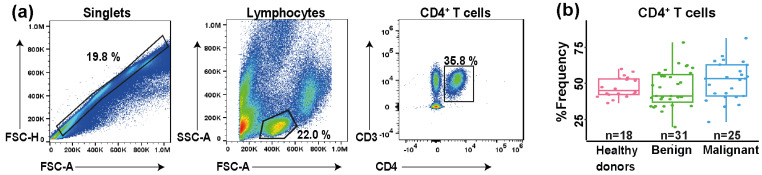
Gating strategy optimization for measurement of CD4^+^ T cells. (**a**) Singlets were selected on an FSC-A/FSC-H plot to exclude doublets and debris. Then, lymphocytes were gated based on FSC-A and SSC-A parameters, and from this gated lymphocyte population, CD3^+^CD4^+^ events were selected on a CD3/CD4 plot. (**b**) Box plot depicting the percentage frequency of CD4^+^ T cells; the percentage values of CD4^+^ T cells were calculated based on total lymphocytes.

The gating strategy was designed to ensure minimal variability and consistent data analysis. First, singlets were selected to exclude doublets and debris. Next, the lymphocyte population was identified based on forward scatter area (FSC-A) and side scatter area (SSC-A) parameters. From this gated lymphocyte population, CD4^+^ T cells were identified by selecting CD3^+^CD4^+^ events (Figure 1a). Additionally, the percentage of frequency of CD4^+^ T cells was compared among the study groups (Figure 1b), revealing no statistically significant differences (*p* = 0.21). However, it is important to note that the percentage of CD4^+^ T cells is more stable in healthy individuals compared to those with tumors. In patients (benign and malignant), the range was 20% to 75%, with a tendency to have more CD4^+^ T cells in those with malignant tumors. In general, this high range may indicate immunological changes (Figure 1b).

### 3.3. Expression of T Cell Senescent Markers on Peripheral CD4^+^ T Cells in Patients with Malignant Ovarian Tumors

**Figure 2:**
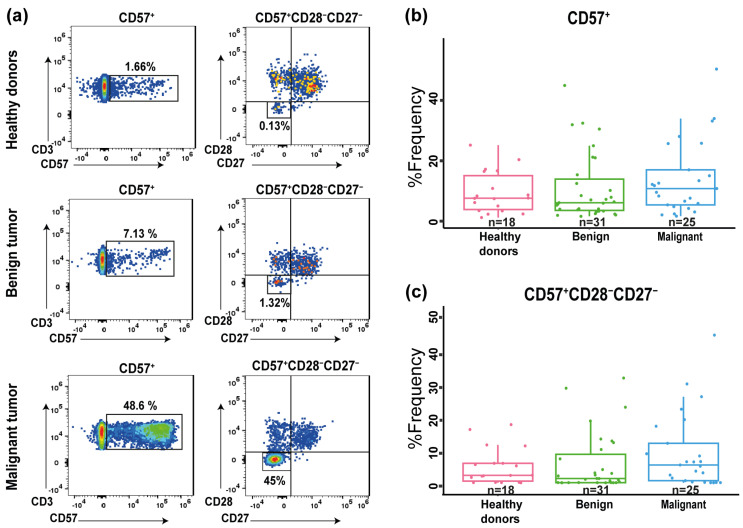
Proportion of senescent CD4^+^ T cells. (**a**) Representative dot plots of CD57^+^ and CD57^+^CD28^−^CD27^−^ populations from healthy donors (n = 18), benign tumor group (n = 31), and malignant tumor group (n = 25) are shown. (**b**) Percentage of circulating CD4^+^ T cells expressing CD57^+^ and (**c**) CD57^+^ CD28^−^CD27^−^ in healthy donors and benign tumor and malignant tumor groups. No significant differences were observed among the groups.

Senescent T cells have undergone extensive activation and proliferation, leading to the loss of key costimulatory molecules, such as CD28 and CD27, while acquiring CD57, a marker of immune exhaustion and reduced proliferative capacity. In the present study, the frequency of CD57^+^CD4^+^ and CD57^+^CD28^−^CD27^−^CD4^+^ T cells were compared among the healthy donors, benign tumors, and malignant tumors (Figure 2). The percentage of frequency of CD57^+^CD4^+^ T cells and CD57^+^CD28^−^CD27^−^CD4^+^ T cells showed a trend toward being higher in the malignant group compared to the healthy donors and benign group, but the difference was not statistically significant (*p* = 0.33 and *p* = 0.22, respectively). Interestingly, both the benign and malignant groups exhibited outliers that might indicate extreme cases of senescent CD4^+^ T cells.

### 3.4. High Expression of Exhaustion Markers on Peripheral CD4^+^ T Cell in Malignant Ovarian Tumors Compared to Benign Tumors and Healthy Donors

**Figure 3:**
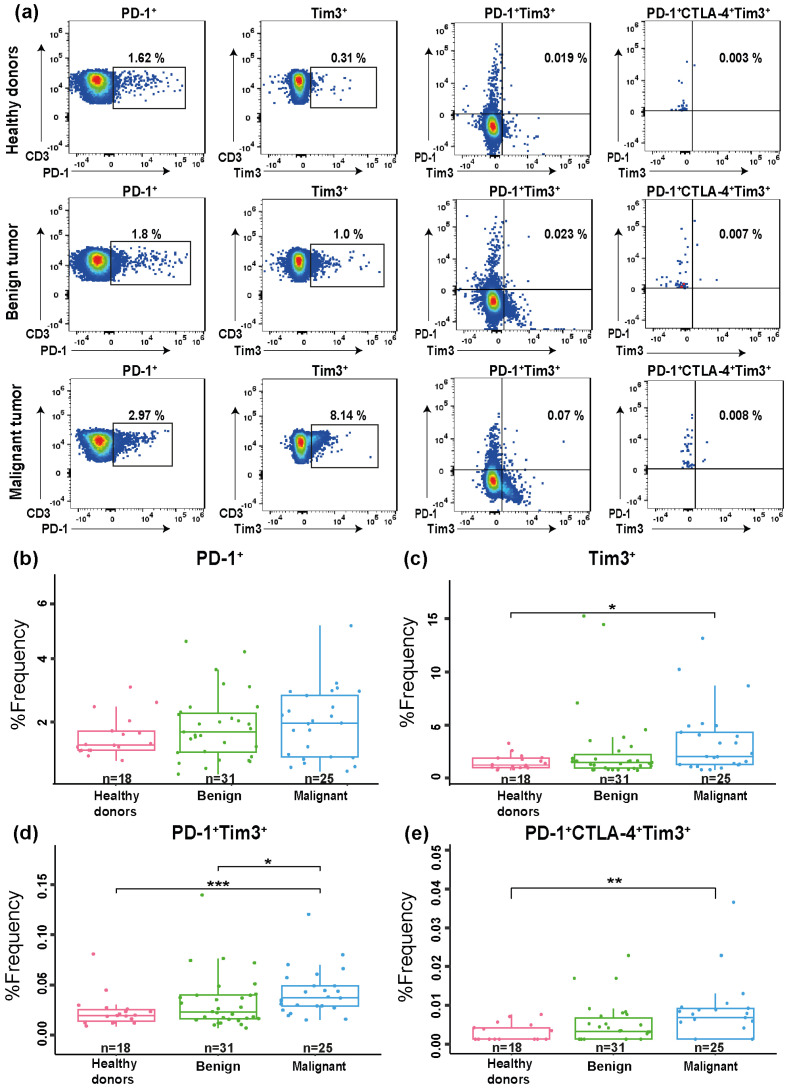
Proportion of exhausted CD4^+^ T cells. (**a**) Representative dot plots of PD-1^+^, Tim3^+^, PD-1^+^ Tim3^+^, and PD-1^+^CTLA-4^+^Tim3^+^ from healthy donors (n = 18), benign tumor group (n = 31) and malignant tumor group (n = 25) are shown. % of frequency of circulating CD4^+^ T cells expressing (**b**) PD-1^+^, (**c**) Tim3^+^, (**d**) PD-1^+^ Tim3^+^, and (**e**) PD-1^+^CTLA-4^+^Tim3^+^ in healthy donors and benign tumor and malignant tumor groups. * *p* < 0.05, ** *p* < 0.01, *** *p* < 0.001.

The frequency of exhausted T cells was analyzed using the individual expression or the co-expression of PD-1^+^, Tim3^+^, and CTLA-4^+^ markers (Figure 3). No significant differences were observed in PD-1^+^ expression on T CD4^+^ cells among groups (*p* = 0.57). However, significant differences were observed in the frequencies of Tim3^+^CD4^+^, PD-1^+^ Tim3^+^CD4^+^, and PD-1^+^CTLA-4^+^Tim3^+^CD4^+^ T cells (*p* = 0.043, *p* = 0.0032, and *p* = 0.016, respectively). In pairwise comparisons, PD1^+^Tim3^+^CD4^+^ expression was significantly higher in the malignant group compared to both the benign group (*p* = 0.05) and healthy donors (*p* = 0.015). Pairwise comparisons showed that Tim3^+^CD4^+^ and PD-1^+^CTLA-4^+^Tim3^+^CD4^+^ expression was significantly higher in the malignant group compared to healthy donors (*p* = 0.039 and *p* = 0.013, respectively).

### 3.5. ROMA Stratification and Its Correlation with Exhaustion Marker Expression in Peripheral CD4^+^ T Cells

**Figure 4:**
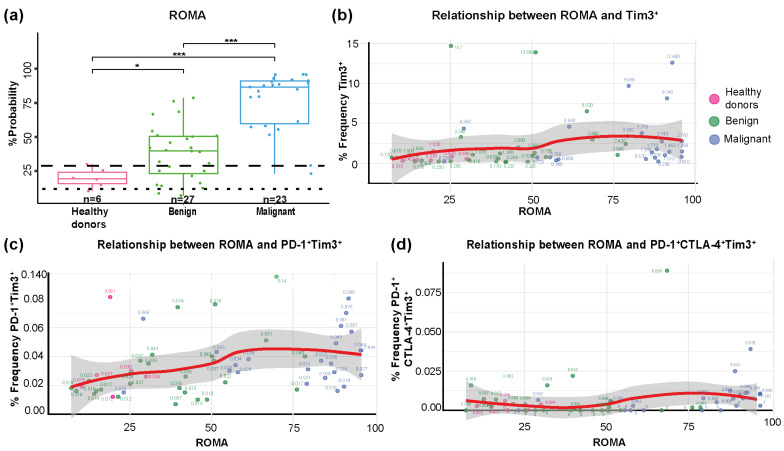
ROMA stratification and association with markers of exhaustion. (**a**) Box plot showing the distribution of ROMA among the three groups (healthy donors, benign, and malignant). Dashed lines indicate the conventional thresholds for a low and a high risk in ROMA probability: 11.4% (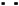) for premenopausal women and 29.9% (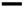) for postmenopausal women. Correlation of ROMA with the percentage frequency of (**b**) Tim3^+^, (**c**) PD-1^+^Tim3^+^, and (**d**) PD-1^+^CTLA-4^+^Tim3^+^ cells. Spearman’s rank correlation was used to determine the correlation coefficient (r). * *p* < 0.05, *** *p* < 0.001.

The Risk of Ovarian Malignancy Algorithm (ROMA) was used to classify patients into low- and high-risk categories for detecting ovarian cancer during ovarian mass surgery. This mathematical calculation incorporates the serum levels of CA-125 (U/mL) and HE4 (pmol/L), as well as the menopausal status (premenopausal or postmenopausal). For premenopausal women, the ROMA index (PI) was calculated using the following formula: PI = exp (−12.0 + (2.38 × ln (HE4)) + (0.0626 × ln (CA-125)). For postmenopausal women, the used formula was as follows: PI = exp (−8.09 + (1.04 × ln (HE4)) + (0.732 × ln (CA-125))). Afterward, the PI was converted into a percentage probability (PP), where PP = exp (PI)/[1 + exp (PI)] × 100, estimating the likelihood of ovarian malignancy. This index is FDA-approved and is used to predict the risk of malignancy in clinical practice. To highlight the importance of ROMA, we compared the values in the three groups (healthy donors n = 6, benign n = 27, and malignant n = 23) using a box plot. A statistically significant difference was observed (*p* = 2.9 × 10^−7^), especially in the malignant group when compared to the healthy donors and benign group (*p* = 2.9 × 10^−5^ and *p* = 8 × 10^−8^, respectively) (Figure 4a). In the box plot, we marked the cut-off values for ROMA at 11.4% (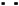) for premenopausal women and 29.9% (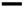) for postmenopausal women. A score below these thresholds indicates low risk (benign), while a score above these values corresponds to high risk (malignant). Using 29.9% as the cut-off value for the Roma algorithm, it demonstrates a sensitivity of 91.3% in classifying patients as malignant. However, the specificity was only 33.3%, as shown in Figure 4a. Therefore, it was of interest to us to evaluate whether the frequency of senescent or exhausted T cells helps improve the sensitivity and specificity of ROMA.

An analysis of the association between the frequency of exhausted CD4^+^ T cells from peripheral blood across the three study groups revealed a positive and significant correlation between ROMA and Tim3^+^ (r = 0.42, *p* = 0.0011) (Figure 4b), as well as with PD-1^+^Tim3^+^ (r = 0.44, *p* = 0.0006) (Figure 4c). A positive correlation was also observed for PD-1^+^CTLA-4^+^Tim3^+^ cells (r = 0.2, *p* = 0.120; Figure 4d), although this was not statistically significant. These findings suggest that PD-1^+^Tim3^+^ expression is significantly associated with ROMA and the presence of malignant tumors, highlighting their potential as key variables for the predictive model.

Given the existing reports on the influence of BMI and age on T cell senescence, exhaustion, and the levels of CA-125 and HE4, three statistical analyses were conducted to evaluate whether BMI and age impact the correlation between ROMA and immune variables (PD-1^+^Tim3^+^ and CD57^+^CD28^−^CD27^−^). First, a multivariable regression model with age as the dependent variable showed a significant association between ROMA and age (β = 0.212, *p* = 0.0001). Second, an ANCOVA model with ROMA as the outcome, adjusted for age and BMI, revealed significant effects of group (*p* = 8.1 × 10^−11^) and age (*p* = 0.000452), but not BMI (*p* = 0.8657). Third, a multiple linear regression model was fitted with ROMA as the dependent variable, and age, BMI, and diagnostic group (benign, malignant) as predictors, using the control group as the reference. The model was significant (R^2^ adjusted = 0.6165) and showed that both age (β = 0.8203, *p* = 5 × 10^−4^) and malignant group (β = 47.8836, *p* = 0) were significantly associated with higher ROMA levels. We observed an association between ROMA and age, which is expected given that ROMA incorporates menopausal status. However, age was not associated with the immune variables, and BMI was not significantly associated with any variables.

### 3.6. Decision Tree Model for Classifying Study Groups Based on ROMA and Frequency of PD-1^+^Tim3^+^

**Figure 5:**
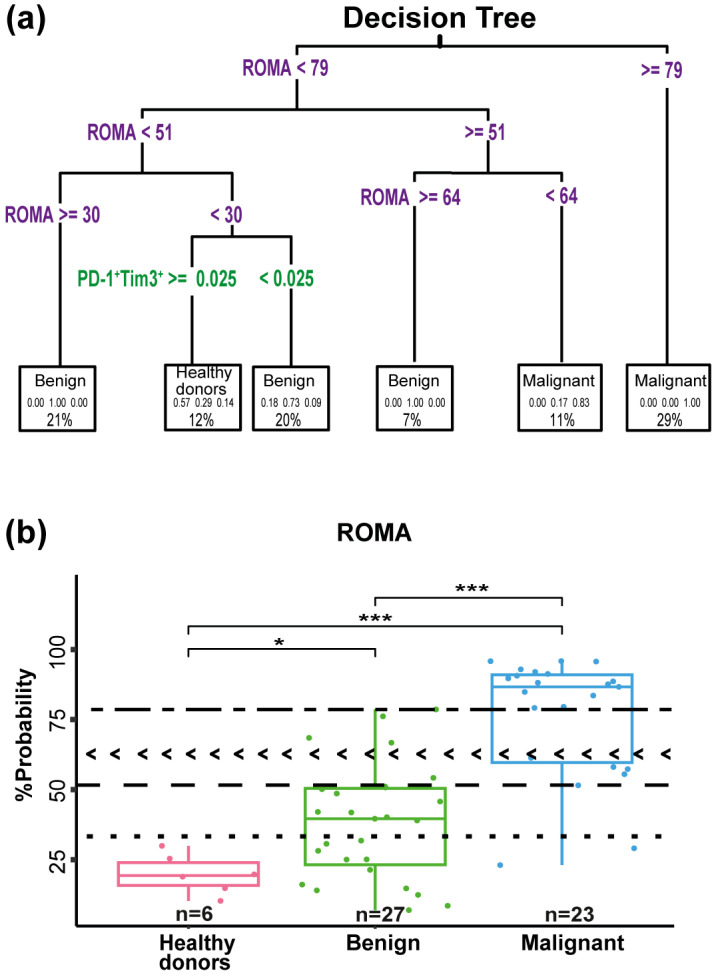
Decision tree based on ROMA and frequency of PD-1^+^Tim3^+^CD4^+^, and ROMA box plot with model-derived cut-offs. (**a**) Decision tree incorporating PD-1^+^Tim3^+^CD4^+^, and ROMA to classify groups, establishing three distinct ROMA cut-off points. (**b**) Box plot with three ROMA classification thresholds derived from the decision tree: low (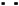), intermediate risk (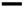), high risk (< <), and extremely high risk (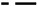). * *p* < 0.05, *** *p* < 0.001.

The distinction between benign and malignant ovarian tumors continues to require careful clinical judgment. Consequently, comparative studies assessing the sensitivity and specificity of routine diagnostic tests are essential for enhancing diagnostic accuracy. In this context, we developed a strategy that incorporates the ROMA parameter with the frequency of PD-1^+^Tim3^+^ on CD4^+^ T cells. The decision tree model (Figure 5a) successfully identified combinations of clinical and immune expression variables associated with the classification of patients into healthy donors, benign, and malignant groups, allowing the identification of new cut-offs for the ROMA.

The first split in the tree was based on the ROMA, with a threshold set at 79. Patients with ROMA ≥ 79 were predominantly classified as malignant. In contrast, patients with ROMA < 79 were directed to a second node, which introduced a new cut-off based on ROMA, with a new threshold set at 51. Patients with ROMA ≥ 51 but <64 were more likely to be classified as malignant, whereas those with ROMA ≥ 51 but ≥64 were likely to be categorized as benign.

Patients with ROMA >51 but ≥30 were directly categorized into the benign group. And the node of <30 was split into two nodes: PD-1^+^Tim3^+^ ≥ 0.025 were categorized as healthy donors and PD-1^+^Tim3^+^ < 0.025 were categorized into the benign group.

Overall, these results demonstrate that ROMA, combined with the frequency of PD-1^+^Tim3^+^, enables the identification of successive risk thresholds, enhancing the differentiation between clinical groups. In the decision tree, we identified four cut-offs at 79%, 51%, 64%, and 30% ROMA and PD-1^+^Tim3^+^. Using these values, we modified the box plot shown in Figure 4b to reflect the new cut-offs, which now classify patients into low, high, and extremely high-risk categories (Figure 5b). With a ROMA above 79%, all patients belonged to the malignant group. In the range between 79% and 51%, some benign patients exhibited behavior similar to that of the malignant group. Between 51% and 30%, most patients were classified as benign, while below 30%, the majority resembled the healthy donor group. Our decision tree model, which uses ROMA and PD-1^+^Tim3^+^ measurements to classify patients, initially appeared to achieve 87.5% accuracy when tested on our study sample of 56 patients. However, this high accuracy was somewhat misleading due to a statistical phenomenon called “optimistic bias”, that is, the model performs better on the same data it was trained on than it would on new patient data.

We used a rigorous statistical technique called bootstrap optimism correction, which involved creating 500 simulated datasets to estimate how much our model was “overfitting” for our specific sample. This analysis revealed that our model had an 11.2% optimistic bias, meaning the true accuracy for new patients would be around 76.3% rather than the initial 87.5%. While 76.3% might seem lower, this represents the model’s genuine diagnostic capability when applied to new patients in clinical practice. Importantly, this performance is still 43 percentage points better than random guessing for distinguishing between the three patient groups (healthy donors, benign, and malignant), indicating that our model has meaningful clinical value for patient classification.

### 3.7. Evaluation of Diagnostic Performance Through Confusion Matrix and ROC Curve Analysis

**Figure 6:**
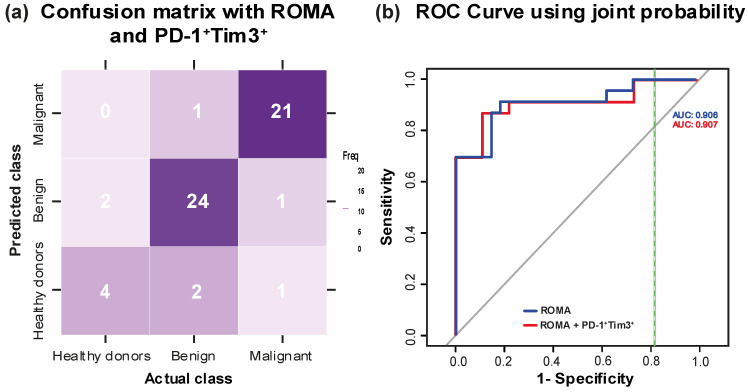
Integrated predictive risk evaluation models. (**a**) Confusion matrix using ROMA and frequency of PD-1^+^Tim3^+^ for the prediction of the three study groups. (**b**) ROC curve using joint probability of ROMA and PD-1^+^Tim3^+^ in the benign and malignant groups.

To evaluate the discriminative performance of ROMA and PD-1^+^Tim3^+^ expression in classifying the study groups (healthy donors, benign, and malignant), we used a confusion matrix based on a classification model trained with these two variables. This allowed us to assess the classification accuracy and the clinical utility of these features for group discrimination. Through the confusion matrix, we can classify the study participants (healthy control n = 6, benign n = 27, and malignant n = 23) based on these predictions. The rows indicate the predicted class and the columns the actual class or the real class (Figure 6a).

This confusion matrix plot shows that the overall classification accuracy attains 87.5%, but with the corrections, the true accuracy for new patients would be around 76.3% rather than the initial 87.5%, indicating that the model correctly classifies 76.3% of the samples. The balanced accuracy for each group was as follows: healthy control 80.4%, benign 89.2%, and malignant 94.1%. Overall, the model demonstrates a good balance between sensitivity (82.2%) and specificity (93.5%) across all classes, indicating its reliability as a tool for classifying the study groups. The kappa coefficient was 0.78, and the confusion matrix reveals that the model achieves good classification accuracy and exhibits substantial agreement between the predicted and actual class memberships, supporting its reliability in distinguishing between the defined groups.

For the ROC curve (Figure 6b), the joint probability of ROMA and frequency of PD-1^+^Tim3^+^CD4^+^ was used, with the benign group as the control and the malignant group as the case. The AUC = 0.907, which, being close to 1, suggests excellent performance. In our analysis, the threshold that maximized the Youden Index was 51.57, with a sensitivity of 86.9% and a specificity of 88.8%, resulting in a Youden Index of 0.758. This indicates that the model effectively differentiates between positive and negative cases, demonstrating a strong balance between sensitivity and specificity and confirming its reliability for distinguishing between classes.

A comparative analysis of the diagnostic performance of ROMA, HE4, CA-125, PD-1^+^Tim3^+^, and their combinations was performed using the ROC curves. Sensitivity, specificity, area under the curve (AUC), and Youden Index values are summarized in Table 2, where cut-off values for the HE4, ROMA, and CA-125 biomarkers were established as follows: 29.9 for ROMA, 35 U/mL for CA-125, and 3510 pg/mL (approximately 140 pmol/L) for HE4 [26]. Although CA-125 individually and in combination with PD-1^+^Tim3^+^ is very effective in confirming the absence of disease, it has limitations in detecting all malignant cases; therefore, it is not the best choice as a primary screening test. In combination, CA-125 + PD-1^+^Tim3^+^ and HE4 + PD-1^+^Tim3^+^, only a slight change in the AUC was observed compared to individual biomarkers. The combination of ROMA and PD-1^+^Tim3^+^ showed the highest Youden Index (0.75) and superior specificity (88.8%) compared with ROMA alone, albeit with a slight decrease in sensitivity (86.9% vs. 91.3%).

Although the HE4 + PD-1^+^Tim3^+^ combination showed slightly higher sensitivity, the ROMA and PD-1^+^Tim3^+^ combination had higher specificity, as well as a higher Youden Index and a similar AUC. This indicates that ROMA + PD-1^+^Tim3^+^ offers a better balance between the ability to correctly detect malignant cases and to avoid false positives; furthermore, the higher AUC reflects a higher overall diagnostic accuracy, making this combination the most robust for discrimination between malignant and benign tumors.

### 3.8. Clinical Nomogram Based on ROMA and PD-1^+^Tim3^+^ to Estimate the Risk of Malignancy

**Figure 7:**
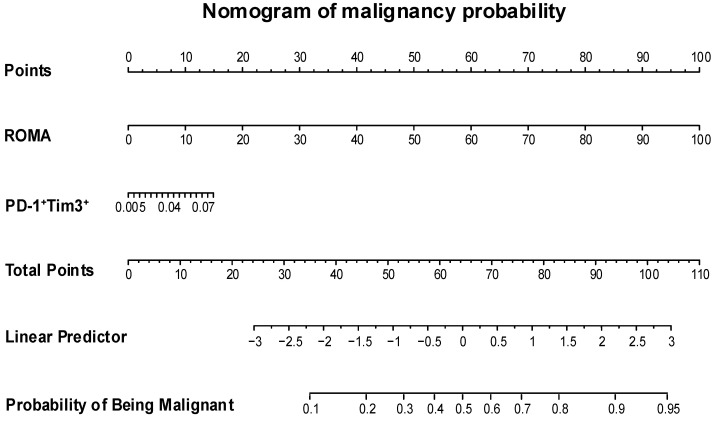
Clinical nomogram using ROMA and PD-1^+^Tim3^+^ to estimate malignancy risk. A nomogram was developed to predict the malignant risk of ovarian tumors, based on the ROMA score and the frequency of PD-1^+^Tim3^+^CD4^+^ T cell.

We designed a nomogram (Figure 7) with the variables and the groups like the ROC curve (malignant and benign). For the design of the nomogram, we perform the complete-case sensitivity analysis, removing the outliers. In the outlier-removal approach, four different detection methods were applied, and only one outlier was removed based on consensus (defined as agreement among ≥2 methods), representing 2% of the total sample (n = 1 out of 50 cases). Their performance was assessed through bootstrap resampling (B = 1000), estimating the optimism-corrected C-index. Internal validation was performed using bootstrap, and model calibration was evaluated by calibration plots and ROC curves. Sensitivity analyses were conducted to assess the robustness of the results across different imputation and outlier handling strategies. The nomogram incorporates independent risk variables obtained from multivariate analysis, effectively predicting the probability of being malignant. This approach demonstrated a high level of discrimination, as indicated by a C-index (Harrell’s concordance index) of 0.91, which means that, in 91% of cases, the model correctly assigns a patient to its corresponding group. The Brier Score of this model was of 0.122, indicating good accuracy of the prediction. For example, if there was a patient with ROMA score 80 (80 points) and frequency PD-1^+^Tim3^+^ was 0.10 (10 points). A total score of 90 corresponds to a malignancy risk of 0.85. As the total score increases, the associated risk of malignancy also rises.

## 4. Discussion

Ovarian cancer (OC) is the most lethal gynecologic malignancy, with a poor prognosis primarily due to late diagnosis and chemoresistance [27]. Screening for ovarian cancer remains challenging due to the lack of effective methods that can reliably detect the disease in its early stages [28]. Diagnosis of ovarian cancer involves a combination of medical history evaluation, imaging tests, and histopathological examination. Front-line treatment typically involves cytoreductive surgery followed by platinum-based chemotherapy [29]. Ovarian tumors can be categorized as either benign or malignant. Benign tumors typically have a favorable prognosis following treatment. However, malignant ovarian tumors are associated with poor clinical outcomes. Consequently, an accurate diagnosis between benign and malignant ovarian tumors is essential for improving patient prognosis and facilitating decision making regarding therapeutic strategies [30]. In this context, the serum levels of CA-125 and HE4 have proven to be valuable tools for the diagnosis of ovarian cancer. These markers help differentiate between malignant and benign tumors. In our study, the levels of both HE4 and CA-125 were significantly elevated in malignant tumors compared to benign tumors and healthy donors, consistent with findings reported in previous research [31,32,33]. Therefore, the most effective diagnostic approach currently appears to be the combination of CA-125 and HE4 levels [8]. Several studies have evaluated the ROMA index, which integrates the serum concentrations of CA-125 and HE4 along with the menopausal status. ROMA has demonstrated utility in distinguishing between benign and malignant pelvic masses with a sensitivity of 0.85 (0.83–0.87) and a specificity of 0.75 (0.73–0.77) [34]. In our study, the ROMA score exhibits a sensitivity of 0.91 and a specificity of 0.37.

However, different patient conditions can bias the ROMA score, for example, it was shown that the ROMA algorithm performs better in postmenopausal women than in premenopausal women [35]. The serum levels of CA-125 and HE4 can also be individually affected by BMI and age [36], conditions that were found to be significantly different between the study groups in this work. A high BMI was observed in patients with benign tumors compared to malignant tumors; this could be associated with early satiety and gastrointestinal symptoms, conditions that are intensified in ovarian cancer patients [37].

Both Treg cells and tumor-infiltrating effector T cells overexpress inhibitory receptors, but with opposite functional consequences: Treg cells maintain their suppressive function, while effector T cells develop dysfunctional phenotypes of senescence and exhaustion [24]. Since CA-125 and HE4 are highly expressed tumor antigens in ovarian cancer, they could contribute to this dysfunctional T cell state and compromise the antitumor response. In this work, we investigate this hypothesis by examining the frequency of exhausted and senescent CD4^+^ T cells in peripheral blood, with the aim of determining whether these dysfunctional immune cell states are associated with ovarian malignant tumors. Previous studies have indicated that immune exhaustion and senescence may contribute to tumor progression [38,39]. Markers of these processes could serve as potential immunological biomarkers to aid in the differentiation between benign and malignant ovarian tumors. Identifying these dysfunctional states in PBMCs is clinically relevant, as they can be easily obtained through minimally invasive blood tests. The detection of immunological signatures in the blood is particularly interesting, as it may reflect a generalized systemic effect on the immune system, suggesting an immunoregulatory response associated with epithelial ovarian cancer [40].

In our study, we show an increased frequency, particularly of CD4^+^ T cells with an exhausted phenotype, in malignant ovarian tumors compared to benign tumors, and in addition, with healthy volunteers. Given this context, the presence of senescent and exhausted T cell phenotypes in peripheral blood may reflect an immunological landscape that favors tumor progression, even in early or non-metastatic stages. These dysfunctional immune subsets could impair effective tumor surveillance and limit the efficacy of antitumor responses. Furthermore, their detection in peripheral blood suggests they may serve as accessible, minimally invasive biomarkers for stratifying ovarian tumor types.

Although the expression of the CD57^+^ marker and the senescence-associated phenotype (CD57^+^CD28^−^CD27^−^) did not reach statistical significance, we observed a trend toward increased frequencies of senescent CD4^+^ T cells. Age-related changes in T cell populations are not solely a consequence of chronological aging but are also shaped by factors such as cumulative antigen exposure and individual genetic and nutritional backgrounds. While CD8^+^ T cells tend to undergo terminal differentiation with age, CD4^+^ T cells appear to follow a more restrained trajectory, preserving a less differentiated memory phenotype [41,42]. For instance, in patients with advanced high-grade serous ovarian cancer (HGSOC), elevated levels of CD57^+^CD8^+^ in ascites but not in peripheral blood were linked to poor prognosis and resistance to chemotherapy [43]. Similarly, in breast cancer, the accumulation of CD4^+^ and CD8^+^ T cells with a senescent phenotype was also reported [44]. Together, these findings highlight the relevance of immune senescence markers as potential indicators of cancer-associated immune dysfunction.

Although our analysis was limited to CD4^+^ T cells, we observed significant associations with the expression of late-exhaustion markers and pelvic mass malignancy. The expression of PD-1, Tim-3, and LAG-3 has been previously observed in ovarian tumor tissues and ascitic fluid [45,46]. Moreover, it has been observed in other malignancies, such as glioblastoma [46]. In addition, changes in the expression of these exhaustion markers were also observed in peripheral blood across different types of cancer, including non-small lung cancer and breast cancer [45].

In this study, we identified positive Spearman correlations between the ROMA scores and the frequency of circulating CD4^+^ T cells expressing Tim3^+^, PD-1^+^Tim-3^+^, and PD-1^+^ Tim3^+^CTLA-4^+^. Among these subsets, the strongest association was observed with PD-1^+^ Tim3^+^ expression. Based on this finding, we integrated the ROMA scores with the frequency of PD-1^+^Tim3^+^CD4^+^ T cells to develop predictive models. This approach was motivated by the limited number of studies addressing multiclass classification strategies for differentiating ovarian tumor types. Currently, ROMA utilizes fixed cut-off values to determine malignancy risk (11.4% for premenopausal and 29.9% for postmenopausal patients) [8,47]. A study conducted in Korean women reported that, in Asian populations, it may be necessary to redefine HE4 and ROMA cut-offs, as HE4 levels tend to increase more significantly with age than with menopausal status [48]. These findings challenge the utility of using dichotomous menopausal-based thresholds and support the adoption of population-specific cut-offs to improve diagnostic precision. Additionally, another study emphasized that the menopausal status, histological subtype, and population demographics should all be considered when defining biomarker thresholds, reinforcing the importance of a personalized approach to diagnosis based on clinical and epidemiological contexts [49]. Some studies have explored modifications to the ROMA algorithm by substituting the menopausal status with patient age. While this adjustment may provide certain advantages, such as improved specificity, the overall diagnostic performance does not appear to be significantly enhanced. In general, both approaches, whether based on age or the menopausal status, yield comparable results in terms of accuracy and clinical utility [8].

The association between the elevated ROMA scores and the increased expression of exhaustion markers on T cells suggests a potential interplay between systemic tumor biomarkers and the immune landscape. High ROMA scores, indicative of a greater likelihood of malignancy, reflect tumor progression and biologic activity. In this context, we hypothesize that the elevated ROMA scores may also be indirectly associated with an immunosuppressive environment, characterized by exhausted CD4^+^ T cells. The integration of immune exhaustion profiling with the ROMA scoring could enhance diagnostic accuracy in distinguishing between malignant and benign tumors. In this regard, considering the ROMA score and the frequency of PD-1^+^Tim3^+^, a decision tree was constructed to distinguish between benign and malignant ovarian tumors. Alternative thresholds were established to better capture the immunological landscape and enhance classification performance. The four new thresholds were 30%, 51%, 64% and 79%. Values below 30% indicate low risk, 30–51% intermediate risk, 64–79% high risk, and above 79% an extremely high risk. Additionally, a value of 0.025 for PD-1^+^Tim-3^+^ was established. Our decision tree model provides a valuable tool to enhance diagnostic precision. The model can be retrained using larger and more heterogeneous patient cohorts, thereby improving its accuracy and broadening its applicability. The prediction probability of this model and the true classification were used to construct a confusion matrix, which assessed the model’s classification performance and evaluated the clinical utility of the selected variables.

We developed and internally validated a nomogram based on clinical and immunological parameters that allows for the more accurate stratification of malignancy risk in patients with ovarian tumors, complementing conventional imaging examinations. This easy-to-use tool has the potential to help clinicians identify patients with malignant tumors and provide patient-specific prognostic information. With further clinical validation, it could serve as a clinically applicable tool, incorporating additional relevant variables to generate a comprehensive risk score.

The advantage of designing predictive models with ROMA and the frequency of PD-1^+^Tim-3^+^ expression in CD4^+^ T cells is that ROMA has high relevance and is widely used in the detection of ovarian cancer. It also uses two biomarkers approved for ovarian cancer detection (HE4 and CA-125). On the other hand, the expression of an exhausted phenotype is specifically associated with cancer or chronic infections, unlike CEA, CA-125, HE4, etc., which can be highly expressed in benign diseases. Another important aspect of PD-1^+^Tim-3^+^ is that it provides a new perspective in immunotherapy (for the design of targeted therapies) and tumor progression, establishing the response to the interaction between cancer and immune cells, and guiding prognosis in patients.

## 5. Conclusions

In conclusion, our study obtained promising results with this novel immunological marker for the diagnosis of pelvic masses; however, further external validation is required to confirm its clinical utility. The combination of the ROMA score with the expression of PD-1^+^ and Tim-3^+^ in CD4^+^ T cells creates a simple yet highly effective model to differentiate between benign and malignant ovarian tumors.

## Figures and Tables

**Table 1:** Clinical characteristics and biomarkers.

	Healthy Donors (n = 18)	Benign (n = 31)	Malignant (n = 25)	Total (n = 74)	*p*
Age (years)	43.22 ± 11.10	44.06 ± 12.36	51.8 ± 7.88	46.47 ± 11.26	0.012 *
BMI	24.83 ± 2.96	29.72 ± 5.34	28.87 ± 5.52	28.22 ± 5.26	0.004 *
Age at menarche	12 (11–13)	12 (11–13.5)	12 (11–13)	12 (11–13)	0.84
CA-125 (U/mL)	19.29 (17–22)	53.2 (19.2–198)	712.10 (273–779)	105.7 (4.4–836)	<0.0001 **
HE4 (pg/mL)	1968 (1758–2217)	2788 (2346–3360)	5214 (3908–6515)	3477(2373–4815)	<0.0001 **
Menopause					
Yes	6 (33.3)	11 (35.5)	16 (64)	33 (44.6)	
No	12 (66.6)	20 (64.5)	9 (36)	41 (55.4)	0.05
Cancer history					
Yes	8 (44.4)	20 (64.5)	17 (68)	45 (60.6)	
No	10 (55.5)	11 (35.5)	8 (32)	29 (39.4)	0.25

BMI (body mass index), CA-125 (cancer antigen 125), and HE4 (human epididymis protein 4). The values are presented as mean ± SD, median (IQR), or number (%). * *p* < 0.05, ** *p* < 0.01.

**Table 2:** Comparative diagnostic performance of HE4, CA125, ROMA, and their combinations with PD-1^+^Tim3^+^ evaluated by the ROC curves.

Variable	Sensitivity (%)	Specificity (%)	AUC	Youden Index	*p*-Value AUC vs. 0.5
* ROMA	91.3%	37%	0.907	0.28	3.74 × 10^−19^
* CA-125	95.7%	29.6%	0.855	0.25	3.2 × 10^−10^
* HE4	91.3%	77.8%	0.88	0.69	8.6 × 10^−14^
PD-1^+^Tim3^+^	82.6%	55.5%	0.682	0.39	0.0183
ROMA + PD-1^+^Tim3^+^	86.9%	88.8%	0.907	0.758	1.04 × 10^−17^
CA-125 + PD-1^+^Tim3^+^	65.2%	96.2%	0.86	0.61	3.87 × 10^−11^
HE4+ PD-1^+^Tim3^+^	91.3%	85.7%	0.89	0.77	9.99 × 10^−15^

* The values of sensitivity, specificity, and Youden Index were calculated using specific cut-offs: 29.9 for ROMA, 35 U/mL for CA-125, and 3510 pg/mL (approximately 140 pmol/L) for HE4. AUC (area under the curve), CA-125 U/mL (cancer antigen 125), HE4 pg/mL (human epididymis protein 4), and ROMA (Risk of Ovarian Malignancy Algorithm).

## Data Availability

The original contributions presented in this study are included in the article. Further inquiries can be directed to the corresponding authors.
